# Diversity, functional traits and assembly processes of diatom community in the aquatic-terrestrial ecotone: a case study of Danjiangkou Reservoir, China

**DOI:** 10.3389/fmicb.2025.1690275

**Published:** 2025-10-31

**Authors:** Xucong Lyu, Haiyan Chen, Jialin Jin, Huatao Yuan, Jing Dong, Yunni Gao, Xiaofei Gao, Jingxiao Zhang, Xuejun Li

**Affiliations:** ^1^College of Fisheries, Henan Normal University, Xinxiang, Henan, China; ^2^Observation and Research Station on Water Ecosystem in Danjiangkou Reservoir of Henan Province, Nanyang, Henan, China; ^3^Ecological Environment Monitoring and Emergency Center of the Source of South-to-North Water Diversion Project in Henan Province, Nanyang, Henan, China; ^4^The National Ecological Quality Comprehensive Monitoring Station (Hebi Station), Hebi, China

**Keywords:** soil diatom, aquatic-terrestrial ecotone, community assembly, community diversity, Danjiangkou Reservoir

## Abstract

Soil diatoms are essential components of microalgae communities in aquatic-terrestrial ecotones, contributing to primary production, organic matter production, nutrient cycling, and ecosystem stability. However, the functional traits and community assembly processes of diatoms in these ecotones remain poorly understood. This study used 18S rDNA high-throughput sequencing and functional trait analysis to investigate the dynamics, driving factors, and assembly processes of diatom communities in the aquatic-terrestrial ecotone of the Danjiangkou Reservoir. Significant differences in diatom diversity and composition were observed between waterward (frequently submerged) and landward zones (exposed to alternating wet and dry conditions). Functional traits revealed that larger, highly motile species dominated waterward communities, while smaller, less motile taxa were prevalent in landward zones. Total phosphorus (TP), soil organic matter (SOM), and magnesium (Mg^2+^) were key environmental drivers of taxonomic and functional traits. Community assembly in both zones was primarily driven by random processes; however, deterministic processes, particularly heterogeneous selection, had a stronger influence in the landward zone, reflecting significant environmental filtering. These findings enhance understanding of biodiversity and community assembly in aquatic-terrestrial ecotones and provide valuable insights into their ecological dynamics.

## 1 Introduction

Reservoir construction provides significant ecosystem services, such as water regulation, flood control, and energy production (Song et al., 2023). However, it also has profound and long-lasting impacts on riverine ecosystems by altering hydrological dynamics, disrupting habitat continuity, and influencing biodiversity (Suen and Eheart, 2006; Vanni et al., 2005). Due to artificial water regulation, reservoirs commonly form aquatic-terrestrial eco-tones characterized by periodic flooding and drying. While such ecotones are also found in natural lakes, rivers, and wetlands, reservoir-driven water level fluctuations can generate pronounced environmental gradients that shape community structure and ecosystem processes (Yin et al., 2018). This zone, despite its instability and ecological vulnerability, provides irreplaceable ecological functions (Shi et al., 2021). As a buffer for regulating the hydrological cycle, it assists in purifying diffuse pollution within the watershed, stabilizing reservoir shorelines, preventing soil erosion, and offering crucial habitats for a variety of species (Xiao et al., 2012; Khurram et al., 2023). Due to its distinctive geographic position and essential ecological functions, the aquatic-terrestrial ecotone is regarded as a critical area for biogeochemical cycling within reservoir systems (Zhang et al., 2022).

Diatoms, as major primary producers, play a crucial role in energy flow and biogeochemical cycles in aquatic ecosystems (Tréguer et al., 2018). They are found not only in aquatic environments but are also common in soils, wetlands, and other habitats thus making them a valuable tool for understanding ecological responses to environmental changes (Riato and Leira, 2020; Pandey, 2020). Diatoms are highly sensitive to variations in water chemistry, nutrient levels, and hydrological conditions; hence, they are important indicators of ecological health (Vazquez et al., 2024; Foets et al., 2020). In aquatic-terrestrial ecotones, diatom functional traits, such as cell size, motility, and attachment (Ács et al., 2020), significantly contribute to ecosystem processes. For instance, larger diatoms enhance organic matter accumulation through higher biomass production, supporting nutrient cycling, while highly motile diatoms optimize light and nutrient acquisition, boosting photosynthetic efficiency (Kahlert et al., 2021). Attached diatoms stabilize substrates, reducing erosion and providing microhabitats for other organisms (Weilhoefer and Pan, 2022). These traits are particularly critical in transitional zones, where periodic flooding and drying create dynamic environmental gradients that select for specific functional adaptations, influencing ecosystem stability and resilience (Gelis et al., 2025). Despite their significance, research on diatoms in aquatic-terrestrial ecotones remains scarce. Most previous studies have concentrated on either fully aquatic or fully terrestrial systems (Zhao et al., 2024a; Ding et al., 2024), resulting in a gap in our comprehension of how diatoms adapt and thrive in the transitional environments characteristic of ecotones. Moreover, while much is known about diatom taxonomy, the functional traits that govern their ecological roles are less thoroughly understood (Virta and Teittinen, 2022; Hu et al., 2023). Functional diversity, which encompasses the range of traits that species exhibit (Wang et al., 2022), is of crucial importance for maintaining ecosystem resilience in the face of disturbances. In aquatic-terrestrial ecotones, where environmental conditions fluctuate due to changes in water levels, understanding the functional diversity of diatom communities can provide insights into how these ecosystems maintain stability.

The mechanisms driving biodiversity formation are closely associated with community assembly processes, which encompass both deterministic processes and stochastic processes (Tripathi et al., 2018; Rojo, 2021). Deterministic processes involve the organization of communities through environmental filtering and biotic interactions, including competition and predation (Wu et al., 2023). In these cases, species are “filtered” into or out of communities according to their ability to adapt to specific environmental conditions. For example, in environments where species share similar niche requirements, strong competition can lead to the convergence of community composition (Hanson et al., 2012). On the contrary, stochastic processes encompass random events such as dispersal, migration, random colonization, and local extinction, which shape community structure independently of species’ ecological traits (Alonso et al., 2006). When stochastic processes dominate, species distribution and abundance are influenced more by chance than by adaptation. The balance between these assembly mechanisms fluctuates with biological scale, habitat complexity, and spatial dimensions (Coffer et al., 2021; Liu et al., 2022). Therefore, gaining insight into the interaction between deterministic and stochastic processes is essential for clarifying the dynamic assembly mechanisms of microbial communities and their responses to environmental fluctuations. However, research on the community assembly mechanisms of diatom communities in aquatic-terrestrial ecotones remains limited. Understanding these processes is essential for uncovering the mechanisms that generate and sustain biodiversity in such dynamic environments.

This study was conducted at the Danjiangkou Reservoir, one of Asia’s largest artificial freshwater reservoirs and a key water source for the middle route of the South-to-North Water Diversion Project (Zhu et al., 2024). The water level of the Danjiangkou Reservoir undergoes significant seasonal fluctuations and is subject to human regulation, especially during the flood and dry seasons (Chen et al., 2020). These fluctuations lead to the formation of pronounced riparian zones, known as drawdown areas. Due to the alternation between inundation and exposure, these areas exhibit distinct ecological characteristics. These transitional zones offer a unique opportunity to study the interactions between aquatic and terrestrial ecosystems (Zhang et al., 2021). In this transitional zone, frequent flooding and drying create a highly heterogeneous environment, which influences species composition and ecological processes. We explored the differences in diatom community composition across different habitats, as well as their trait distribution and ecological processes. (1) How do diatom community composition and diversity differ between waterward and landward environments? (2) What factors influence the distribution of diatom traits in waterward and landward environments? (3) What ecological processes govern diatom community assembly in the soil of the aquatic-terrestrial ecotone? This study aims to provide insights into how environmental variability drives diatom community structure in dynamic ecotonal regions.

## 2 Materials and methods

### 2.1 Study area

The Danjiangkou Reservoir, located in central China across Hubei and Henan provinces, is one of the country’s largest artificial lakes (Chen M. et al., 2022). Built in 1958, it serves multiple functions, including flood control, irrigation, power generation, and urban water supply (Chao et al., 2019). With a total capacity of 29 billion cubic meters and a surface area spanning several hundred square kilometers, the reservoir is located in a region that experiences a humid climate with distinct seasons and abundant annual rainfall. It is primarily fed by the Danjiang and Hanjiang rivers, thus creating a complex hydrological environment (Chen Z. J. et al., 2022).

**Figure 1:**
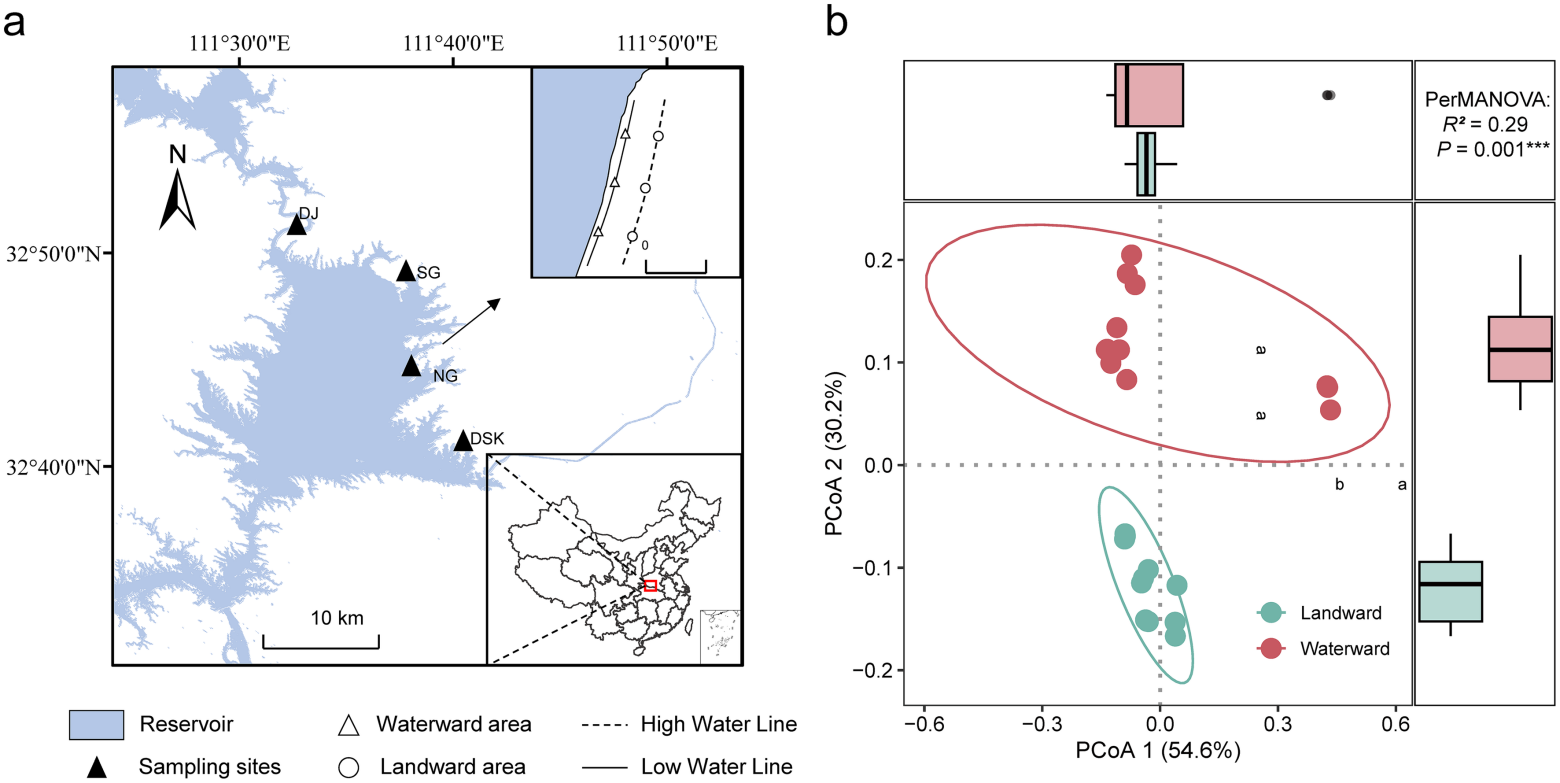
**(a)** Sampling sites in the Danjiangkou Reservoir; **(b)** The PCoA of environmental factors. Different letters indicate significant differences (*p* < 0.05).

In the study, four main regions within the Danjiangkou Reservoir namely Danjiang (DJ), Diaoshuikou (DSK), Nangang (NG), and Songgang (SG) were selected as sampling sites (Figure 1a; Supplementary Table S1). Sampling was conducted in April 2024, during the dry season when the water level was at its annual lowest. This allowed for maximum exposure of the riparian zone and facilitating the study of diatom community distribution and functional characteristics under varying hydrological conditions. The distance between the high waterline and the low waterline was approximately 150 meters.

### 2.2 Sample collection and soil physicochemical properties analysis

In April 2024, samples were collected from the waterward and landward areas of the Danjiangkou Reservoir. A total of 24 sampling sites were chosen from four regions, with three sites each along the high waterline (landward locations, with an average annual inundation period of 320 days) and three sites along the low waterline (waterward locations, with an average annual inundation period of 150 days and an exposure period exceeding 45 days prior to sampling) in each region. At each sampling site, surface soil (0–5 cm) was collected using a soil corer after removing visible plant roots and stones. The samples were promptly frozen at −20 °C and transported to the laboratory for chemical and DNA analyses. For chemical analysis, the soil samples were freeze-dried and passed through a 2 mm sieve to eliminate large debris, then stored at 4 °C until further examination. Subsamples intended for soil DNA extraction were stored at −80 °C to maintain DNA integrity.

Nitrate nitrogen (NO₃^−^-N) and ammonium nitrogen (NH₄^+^-N) were determined through spectrophotometric methods. Total phosphorus (TP) and total nitrogen (TN) were measured through acid digestion methods. Soluble calcium ions (Ca^2+^) and magnesium ions (Mg^2+^) were analyzed with atomic absorption spectrophotometry. The content of Iron (Fe) content was determined through colorimetric methods. Soil organic matter (SOM) was measured by the Walkley-Black method, while soil moisture content (SMC) was calculated through oven-drying.

### 2.3 DNA extraction and Illumina sequencing

Following the manufacturer’s instructions, total DNA was extracted from soil samples using the DNeasy PowerSoil Pro Kit (Qiagen, CA, United States). The V4 hypervariable region of the 18S rRNA gene was amplified with barcode primers DIV4for (5′-GCGGTAATTCCAGCTCCAA-3′) and DIV4rev (5′-AATCCRAGAATTTCACCTCT-3′) (Malviya et al., 2016). The construction of the gene library followed the procedure described by Liu et al. (2019). PCR products from all samples were pooled in equimolar amounts, and after quality control, sequencing was performed on the Ion S5™ XL platform (Thermo Fisher Scientific) by Novogene Co., Ltd. (Beijing, China).

Paired-end reads were assigned to samples using unique barcodes and processed by removing barcode and primer sequences. FLASH (v.1.2.11) (Magoc and Salzberg, 2011) was used to merge overlapping paired-end reads, generating raw tags. Quality filtering was performed with fastp (v.0.23.1) to obtain high-quality clean tags (Bokulich et al., 2013). Chimera sequences were identified and removed by comparing tags against the Silva reference database (v.138.2) using Vsearch (v.2.16.0) (Edgar et al., 2011), resulting in the final effective tags. Additional details on sequencing and taxonomic assignment are available in Wang et al. (2019).

### 2.4 Identification of diatom function traits

To assess the functional diversity of diatoms in the aquatic-terrestrial ecotone of the Danjiangkou Reservoir, we selected functional traits based on their relevance to the ecological roles of diatoms in dynamic transitional environments. Five categories of functional traits were chosen: cell size, Biological Condition Gradient (BCG), motility, attachment, and habitat type. These traits were selected because they directly influence diatoms’ survival, growth, and ecological functions in the context of fluctuating hydrological and nutrient conditions characteristic of the ecotone. Cell size affects resource acquisition and biomass accumulation, with larger cells contributing to higher organic matter production, supporting nutrient cycling. The BCG reflects species’ sensitivity to environmental stressors, such as nutrient gradients and water level fluctuations, which are prevalent in transitional zones. Motility enables diatoms to optimize light and nutrient acquisition through migration, particularly in frequently inundated waterward zones. Attachment traits, such as prostrate or vertical forms, enhance substrate stabilization and resistance to erosion, crucial for maintaining habitat integrity in dynamic environments. Habitat type indicates diatoms’ adaptations to benthic, planktonic, moist, or soil conditions, reflecting their ability to thrive under alternating wet and dry cycles in the ecotone.

Traits essential for the survival, growth, or reproduction of diatoms were compiled from existing literature sources (Wagenhoff et al., 2013; Witteveen et al., 2020). Five categories of functional traits were considered for each species: cell size, Biological Condition Gradient (BCG), motility, attachment, and habitat. Cell size was classified based on cell volume into five groups: nano (0–99 μm^3^), micro (100–299 μm^3^), meso (300–599 μm^3^), macro (600–1,499 μm^3^), and large (≥ 1,500 μm^3^). The Biological Condition Gradient (BCG) was classified in accordance with species sensitivity to environmental changes into specialist species, highly sensitive species, sensitive species, indiscriminate species, and tolerant species. Motility was categorized into five levels: highly motile, moderately motile, slightly motile, weakly motile, and non-motile. Attachment was divided into prostrate, unattached, and vertical. Finally, habitat types were classified into benthic, planktonic, moist habitats, and soils. The detailed classifications of these traits are provided in Supplementary Table S2.

### 2.5 Bioinformatic and statistical analysis

All statistical analyses were conducted in R (v.4.1.2). Taxonomic diversity metrics, including species richness (Richness), Shannon diversity (Shannon), Simpson diversity (Simpson), and Pielou’s evenness (Pielou), were computed using the “vegan” package (v.2.6–2) (Oksanen et al., 2022). Functional diversity metrics, including functional richness (FRic), functional evenness (FEve), functional divergence (FDiv), and the Rao index (RaoQ), were calculated via the “FD” package (v.1.0–12) (Laliberte and Legendre, 2010) based on species traits and taxonomic data. Differences in environmental variables indices between waterward and landward sites were assessed using principal coordinate analysis (PCoA) based on Euclidean distance. Permutational multivariate analysis of variance (PERMANOVA) was conducted via the adonis function in the “vegan” package to test for statistical differences between groups.

To identify key drivers of benthic diatom taxonomic diversity (TD) and functional diversity (FD), multiple linear regression models were applied. Variance inflation factor (VIF) analysis, conducted via the vifstep function in the “usdm” package (v.2.1–5), ensured all predictor variables had VIF values below 10 to mitigate collinearity (Naimi et al., 2013). Model selection was performed using the Akaike Information Criterion (AICc) via the stepAIC function in the “MASS” package (v.7.3.55), with the lowest AICc value determining the optimal model. Prior to regression, environmental variables and diversity indices were log (x + 1) transformed for comparability across models (Guo et al., 2020; Schielzeth, 2010; Wijewardene et al., 2021).

To explore associations between species traits and environmental variables, R-mode linked to Q-mode (RLQ) analysis and the fourth-corner method were employed (Baty et al., 2013). RLQ analysis summarizes the joint structure between environmental, abundance, and trait matrices through multivariate techniques, while the fourth-corner method tested bivariate trait-environment relationships through permutation tests. The False Discovery Rate (FDR) method was applied for multiple testing correction, and RLQ analysis was conducted using the RLQ function in the “ade4” package (v.1.7–18) (Dray et al., 2014; Dolédec et al., 1996).

Using the modified stochasticity ratio (MST) to evaluate the relative importance of deterministic and stochastic processes in community assembly, an MST value < 0.5 indicates that deterministic processes dominate, while an MST value > 0.5 suggests that stochastic processes dominate (Ning et al., 2019). Additionally, the “iCAMP” package (1.5.12) was used to quantify community assembly processes (Ning et al., 2020). The beta taxon index (βNTI) and Raup-Crick index (RCBray) were calculated based on a null model framework. |βNTI| > 1.96 indicated deterministic assembly (βNTI < −1.96: homogeneous selection; βNTI > 1.96: heterogeneous selection), whereas |βNTI| < 1.96 suggested stochastic processes. Within stochasticity, RCBray < −0.95 signified homogenizing dispersal, RCBray > 0.95 indicated dispersal limitation, and |RCBray| < 0.95 represented drift, encompassing weak selection, weak dispersal, diversification, and ecological drift (Stegen et al., 2013).

## 3 Results

### 3.1 Soil physicochemical properties

The PCoA analysis results demonstrated that there were significant differences in environmental factors between the waterward and landward zones (Figure 1b). Owing to the long-term influence of flooding, the soil moisture content (SMC) at waterward sites was markedly higher than that at landward sites. The concentration of iron ions at landward sites was significantly greater than that at waterward sites. The concentrations of nitrate nitrogen (NO₃^−^-N), ammonium nitrogen (NH₄^+^-N), and soluble calcium ions (Ca^2+^) were significantly higher in waterward sites than those in landward locations (*p* < 0.05), respectively. Although the total nitrogen (TN), total phosphorus (TP), and magnesium ion (Mg^2+^) concentrations appear higher in waterward locations, these differences were not statistically significant (*p* > 0.05). The content of soil organic matter (SOM) content showed no significant difference between the two locations (*p* > 0.05). In general, the results indicated that the waterward locations had significantly higher levels of certain nutrients and moisture (Supplementary Figure S1).

### 3.2 Taxonomic and functional structure of diatoms community

Using high-throughput amplicon sequencing, we obtained 6,820,100 reads from the samples, which were grouped into 5,740 ASVs. A total of 102 diatom species were identified. The detailed functional trait classifications for each species are provided in Supplementary Table S4. The composition of the diatom community exhibited variation in accordance with changes in habitat. The results indicated that at the landward sites, the species *Hantzschia amphioxys* (54.70%) and *Cymatopleura elliptica* (9.70%) had relatively higher abundances. Conversely, at the waterward sites, *Cymatopleura elliptica* (20.97%) and *Gyrosigma acuminatum* (12.06%) were the most abundant species (Supplementary Figure S2). At the DSK landward sites (DSK_L1-L3), *Hantzschia* was the overwhelmingly dominant, whereas at the NG waterward sites (NG_W1-W3), *Cymatopleura* was the overwhelmingly dominant genus.

**Figure 2:**
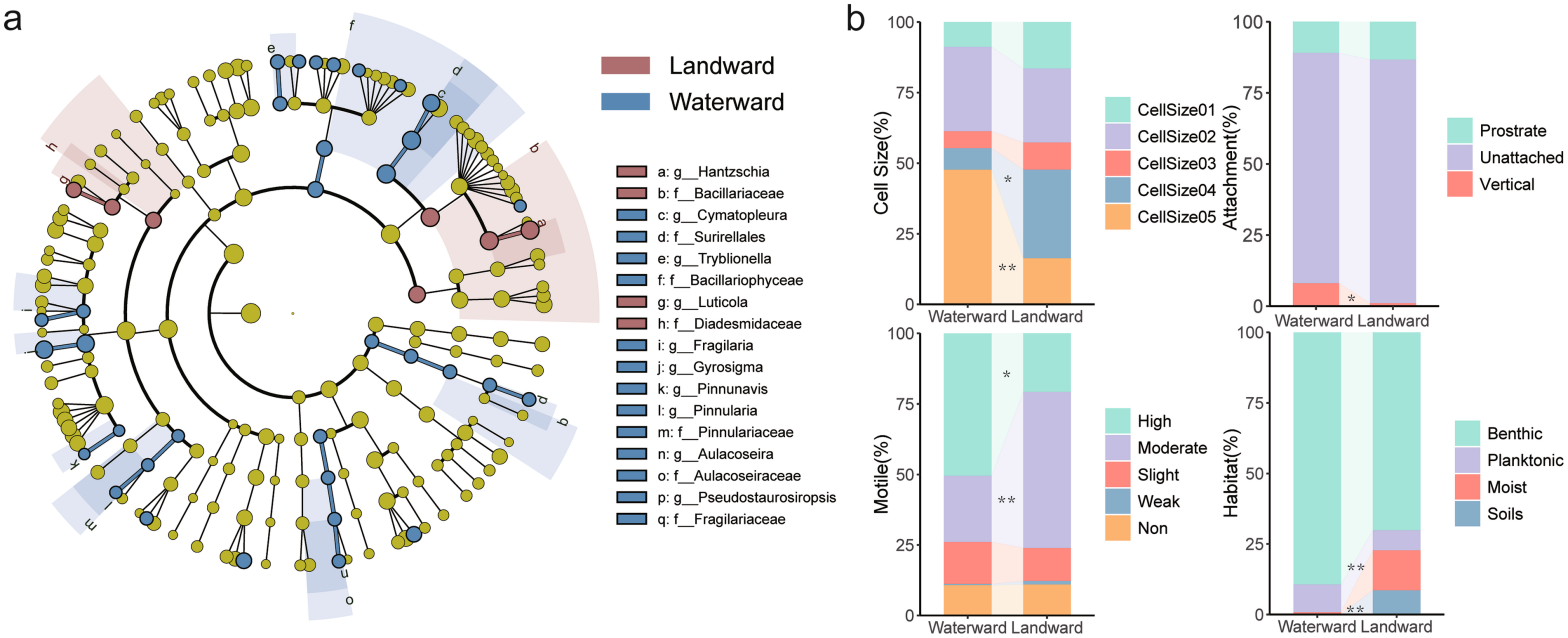
**(a)** The Lefse analysis of diatom communities in waterward and landward; **(b)** Comparison of diatom traits between nearshore and inland areas. The Kruskal-Wallis test was used to assess differences between different sites, *p < 0.05, **p < 0.01.

According to the cladogram results from the LEfSe analysis (Figure 2a), there were significant taxonomic disparities in diatom communities between the waterward area (blue) and landward area (red). In the blue region, species from waterward areas were more abundant in specific families and genera, such as Bacillariaceae, Fragilariaceae, and Aulacoseiraceae. Genera like *Cymatopleura* and *Aulacoseira* exhibited higher significance. On the other hand, landward areas were represented by more species from the *Hantzschia* and *Luticola* genera, as well as the Diadesmidaceae family.

The functional traits of benthic diatoms exhibited significant variations between waterward and landward regions (Figure 2b; Supplementary Table S3). In waterward sites, large-sized diatoms dominated, promoting nutrient cycling through high biomass, while landward sites were characterized by smaller macro cells, adapted to drought. Biological Condition Gradient (BCG) showed no significant difference, indicating similar environmental sensitivity across zones. Highly motile diatoms prevailed in waterward sites, optimizing light and nutrient acquisition, whereas moderately motile diatoms in landward sites conserved energy under fluctuating conditions. Prostrate attachment in waterward sites stabilized substrates, while unattached forms in landward sites suited alternating wet-dry cycles. Benthic diatoms dominated waterward zones, whereas species adapted to moist and soil habitats were more numerous in landward zones, reflecting drought tolerance. These trait differences contribute to greater functional diversity in waterward zones, enhancing resilience to hydrological disturbances, while landward zones exhibit more uniform traits, promoting community stability.

### 3.3 Taxonomic and functional diversity of diatoms and their key driving factors

**Figure 3:**
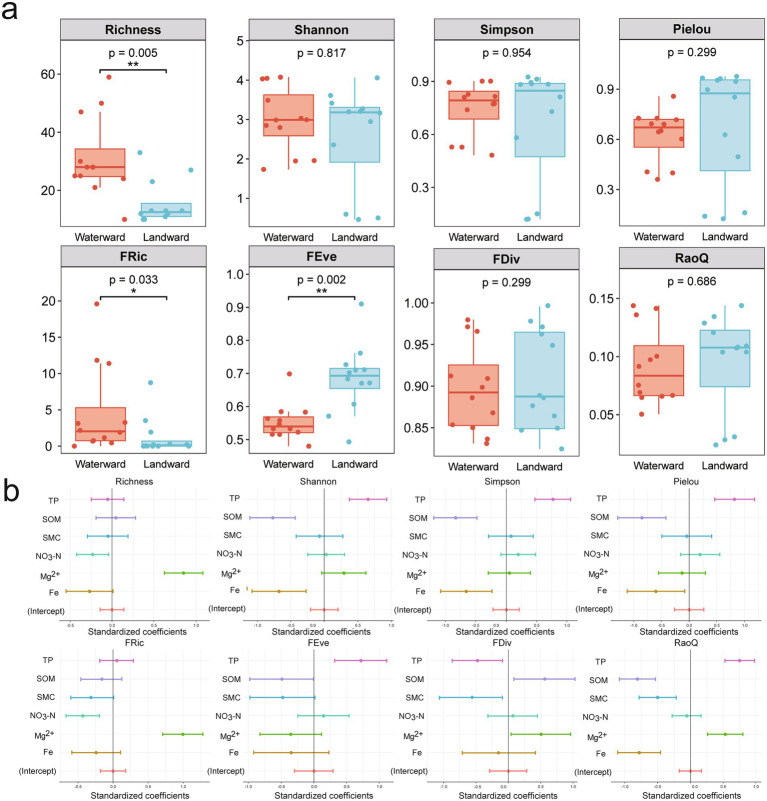
**(a)** Box plots of taxonomic (Richness, Shannon, Simpson, Pielou) and functional (Fric, FEve, FDiv, RaoQ) diversity indexs for waterward and landward sites. **(b)** Standardized regression coefficients and 95% confidence intervals were used to assess the influence of predictors on taxonomic and functional diversity.

The diversity indices exhibited distinct responses between waterward and landward sites. In terms of taxonomic diversity indices, only the Richness index demonstrated a significant difference (Figure 3a). This higher Richness in waterward sites likely reflects greater niche availability due to stable hydrological conditions. Other indices (Shannon, Simpson, Pielou) did not exhibit significant differences (Figure 3a). Regarding functional diversity indices, the FRic index was significantly higher at waterward sites, while the FEve index was significantly higher at the landward sites. Higher FRic suggests enhanced functional adaptability in waterward zones, whereas higher FEve indicates greater community stability in landward zones under fluctuating conditions. No significant differences were observed for the other functional diversity indices.

Multiple linear regression models were further applied to explore the continuous response relationships between environmental variables and diversity patterns. This approach helps to identify potential ecological drivers influencing both taxonomic and functional diversity. Overall, the taxonomic characteristics of the diatom communities were significantly influenced by TP, SOM, and Fe, while the functional traits were significantly affected by TP, SOM, and Mg^2+^ (Figure 3b). Richness exhibited a positive association with Mg^2+^ but a negative one with NO₃^−^. Shannon, Simpson, and Pielou’s evenness indices were positively linked to TP while negatively related to SOM and Fe. FRic displayed a positive correlation with Mg^2+^ and a negative correlation with NO_3_^−^. FEve was positively associated with TP, whereas FDiv showed a negative correlation with TP and SMC but a positive correlation with SOM and Mg^2+^. RaoQ was positively correlated with TP and Mg^2+^ while negatively associated with SOM, SMC, and Fe. In conclusion, diatom taxonomic and functional diversity are significantly influenced by soil physicochemical factors. Specific environmental factors exerted varying effects on different aspects of diversity.

### 3.4 Responses of diatoms traits to environmental factors

**Figure 4:**
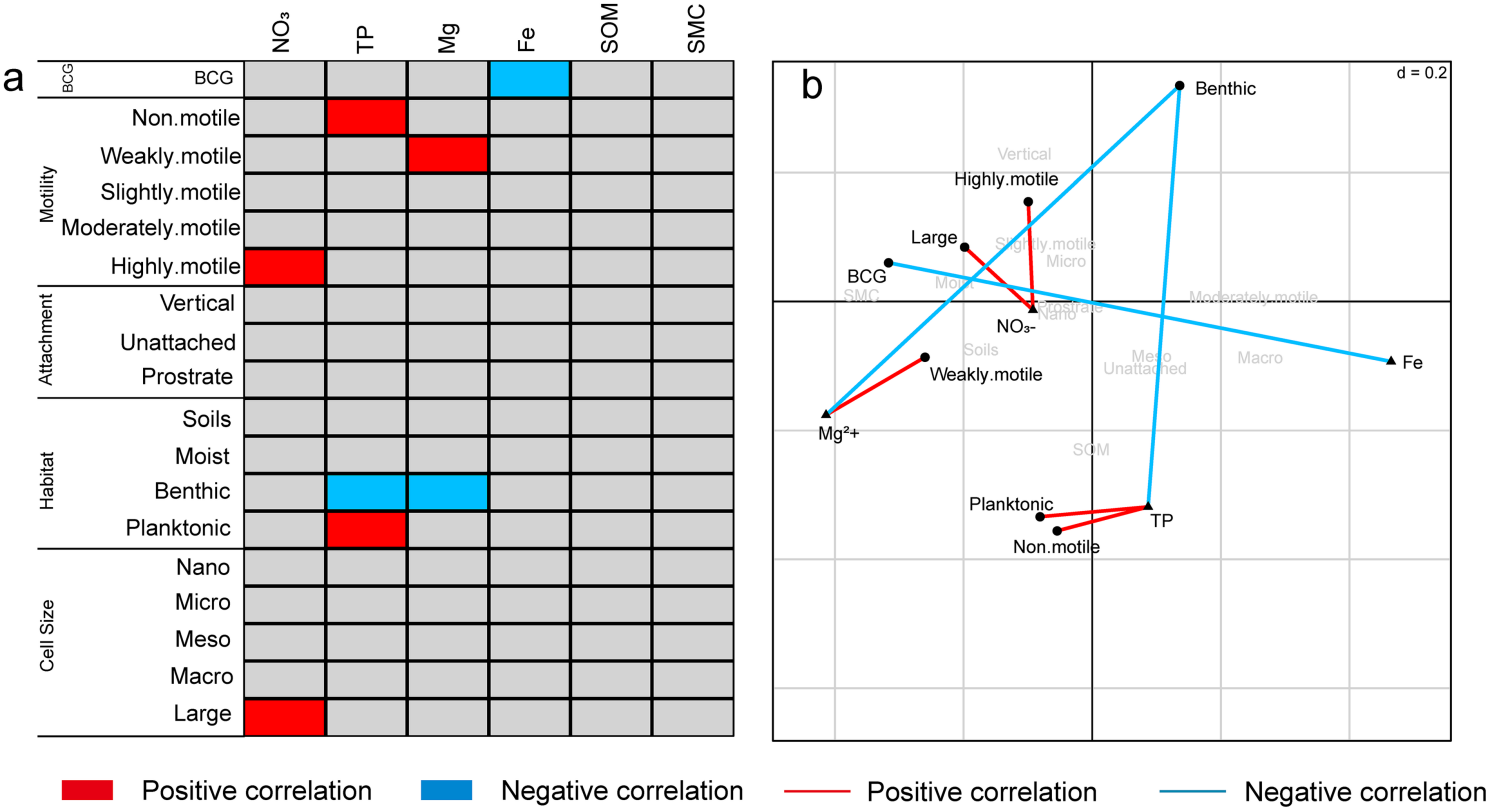
**(a)** The bivariate fourth-corner relationships between species traits and environmental variables. **(b)** Biplot from RLQ analysis showing the relationships between species traits and environmental variables.

The relationships between diatom traits and environmental factors were analyzed using RLQ and the fourth-corner method (Figure 4). BCG exhibited a negative correlation with Fe. Motility was linked to nutrient availability, with non-motile diatoms positively associated with TP, weakly motile diatoms correlated with Mg^2+^, and highly motile diatoms linked to NO^3−^. Regarding habitat preferences, planktonic diatoms were positively correlated with TP, while benthic diatoms were negatively correlated with both TP and Mg^2+^. For cell size, only large diatoms were positively correlated with NO₃^−^. Overall, this analysis demonstrates that environmental gradients, particularly nutrient content (TP, Fe, and Mg^2+^), significantly shape diatom trait distributions, influencing their motility, attachment, and habitat preferences.

### 3.5 Diatom community assembly mechanisms

**Figure 5:**
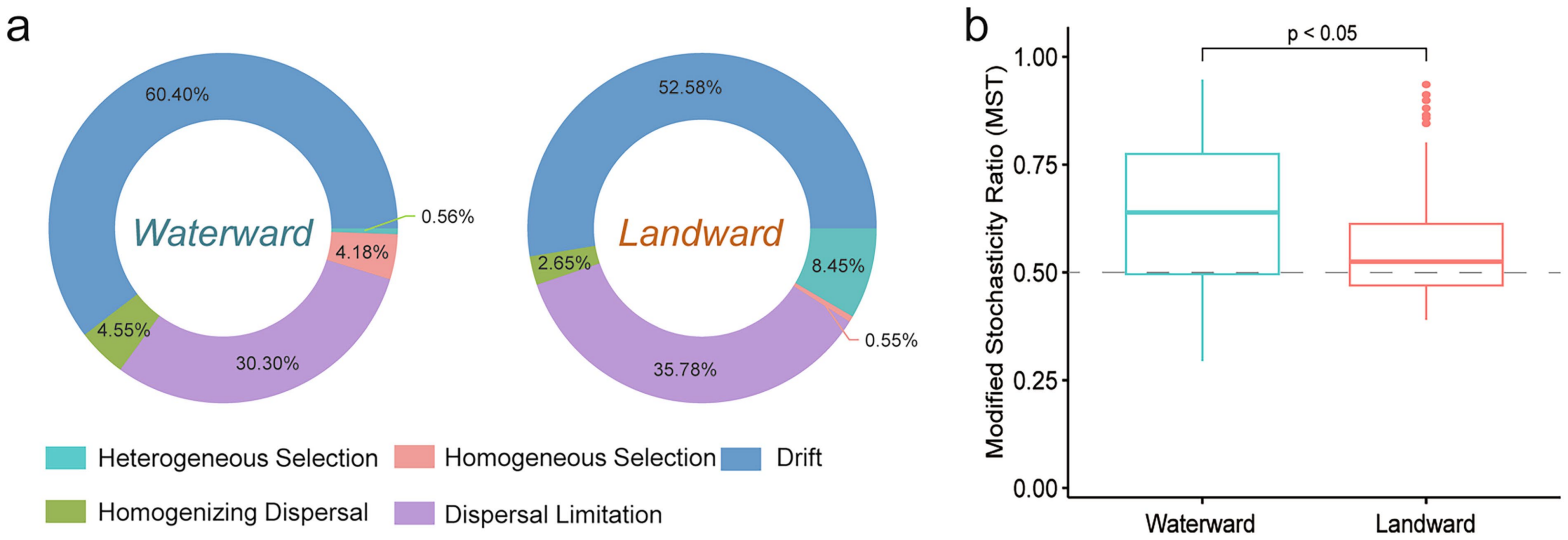
Community assembly mechanisms in waterward and landward sites. **(a)** Show the relative contributions of various ecological processes in the waterward and landward sites, and **(b)** show the percentage of stochastic and deterministic processes based on modified stochasticity ratio.

Community assembly is crucial for understanding the factors influencing taxonomic diversity and abundance in ecological communities. The modified stochasticity ratio (MST) was used to assess the relative importance of deterministic and stochastic processes in diatom community assembly (Figure 5b). The results showed that the MST values in both waterward and landward zones exceeded the 50% threshold, with the waterward zone exhibiting significantly higher values than the landward zone (*p* < 0.05). This indicates that both zones are predominantly driven by stochastic processes, with the waterward zone being more strongly influenced by stochasticity.

We further employed iCAMP to quantify the assembly processes of diatom communities in waterward and landward sites. The results suggested that in the waterward location, 60.54% of community variation was driven by drift, while dispersal limitation and homogenizing dispersal accounted for 30.30 and 4.55%, homogeneous selection and heterogeneous selection accounted for 4.18 and 0.56% (Figure 5a). In contrast, in the landward location, drift remained the dominant factor at 52.58%, while dispersal limitation and homogenizing dispersal accounted for 35.78 and 2.65%, but heterogeneous selection increased to 8.45%, with homogenizing selection accounted for 0.55% (Figure 5a; Supplementary Figure S3). This suggests that while drift is the primary driver in both environments, stochastic processes are more pronounced in the waterward location, whereas heterogeneous selection plays a larger role in the landward location. Overall, the waterward zone communities are more influenced by stochastic processes, while the landward zone experiences a higher degree of deterministic processes, particularly heterogeneous selection.

## 4 Discussion

### 4.1 Diatom community adaptations and functional trait variations in aquatic-terrestrial ecotones

The unique hydrological conditions of aquatic-terrestrial ecotones, which are characterized by periodic flooding and drying, offer a distinct environment that supports diverse diatom communities (San-José et al., 2010). The multifunctionality of ecosystems in transitional environments like aquatic-terrestrial ecotones promotes diatom diversity, particularly because of the availability of both aquatic and terrestrial ecological niches. This dual-niche availability allows species with varying environmental tolerances to coexist, contributing to increased biodiversity (Boyd, 2019; Aranguren-Gassis et al., 2019). In this study, the spatial distribution of diatom species in the Danjiangkou Reservoir revealed significant differences between waterward and landward sites (Supplementary Figure S2). Specifically, *Hantzschia amphioxys* and *Cymatopleura elliptica* dominated the landward and waterward zones, respectively, which is consistent with prior research on transitional habitats (Serieyssol, 2012). Antonelli et al. (2017) found that *Hantzschia* exhibits higher drought tolerance, whereas *Cymatopleura* is more widely distributed in humid or aquatic environments. This is consistent with the results of this study. The significant differences in taxonomic composition between these zones, as confirmed by Lefse analysis (Figure 2a), demonstrate the influence of environmental filtering. Environmental filtering has been shown to play a pivotal role in shaping community composition in ecotonal regions, where species are filtered based on their ability to adapt to rapid environmental fluctuations.

The functional traits of diatoms, including cell size, and motility, play a crucial role in their adaptation to different environmental conditions (Litchman and Klausmeier, 2008; Violle et al., 2007). Previous studies have shown that cell size was closely related to how diatoms perform in terms of resource acquisition, environmental stability, and predation pressure (Edwards et al., 2013; Ruwer and Rodrigues, 2022). Larger cells tend to store more nutrients, making them better suited to nutrient-rich aquatic environments, while smaller cells are more adaptable to resource-scarce or unstable conditions (Klip et al., 2024). In terms of motility, highly motile species can more effectively seek out optimal habitats and avoid unfavorable conditions, such as sedimentation or low light (Morelle et al., 2024; Pandey and Bergey, 2016; Abbriano, 2023). In this study, significant differences were observed in the distribution of diatom traits between the waterward and landward zones (Figure 2b). These findings align with and expand upon earlier research by demonstrating how diatom traits vary across environmental gradients and suggesting specific mechanisms underlying their adaptation. In the waterward zone, the diatom communities were dominated by large cells, and highly motile species. These traits suggest that waterward diatoms benefit from the stable nutrient supply and moist conditions of the aquatic environment. Large cells can store more resources in the nutrient-rich conditions, while high motility allows diatoms to efficiently migrate to areas with optimal light for photosynthesis (Wu et al., 2024). In contrast, the landward zone was characterized by smaller cells, and moderately motile species. This indicates that landward diatoms are better adapted to drier conditions and lower nutrient availability, where smaller cell size helps reduce water loss (Hansen and Visser, 2019). These trait differences can be attributed to the selective pressures imposed by the environmental gradients in different habitats (Gómez et al., 2024). In the waterward zone, stable hydrological conditions and nutrient levels favor the development of larger cells and stronger motility to maximize resource utilization. In the landward zone, frequent drying and lower nutrient availability select for diatoms with smaller cells, as these traits allow for better adaptation to environmental fluctuations, and reduced water loss.

### 4.2 The driving factors of diatom community diversity and functional traits

Functional diversity, which reflects the range of traits that organisms possess to perform ecological roles (Gozdziejewska et al., 2024), is crucial for maintaining ecosystem stability, particularly in dynamic environments like aquatic-terrestrial ecotones (Zhao et al., 2024b). While previous research on diatoms has largely focused on taxonomic diversity, more recent studies have highlighted the importance of functional diversity in understanding how ecosystems respond to environmental fluctuations (Kamberovic et al., 2024). The functional richness (FRic) in the waterward zone is significantly higher than that in the landward zone, suggesting that diatom communities in this region are required to adapt to frequent water level fluctuations and a consistently humid environment. Such highly dynamic conditions foster increased functional diversity by facilitating species coexistence mechanisms. In contrast, the higher functional evenness (FEve) observed in the landward zone indicates a relatively stable community structure, with smaller functional differences among species (Figure 3a). Studies had shown that functional traits determined how diatoms adapted to varying environmental conditions, including nutrient availability, moisture levels, and hydrological changes (Virta and Teittinen, 2022). These traits significantly influence diatom survival and community composition in environments subject to abiotic stress (Gill et al., 2022). This study also confirms this, showing that fluctuating environments, such as those in the waterward zone, may enhance functional trait diversity by increasing niche opportunities and reducing competitive exclusion among species (Gill et al., 2022; Terseleer et al., 2014).

Soil physicochemical factors, including nitrate nitrogen (NO₃^−^-N), calcium ions (Ca^2+^), and iron (Fe), exert significant influence on diatom functional traits and community functional diversity (Terseleer et al., 2014). Numerous studies have demonstrated that nutrient levels not only affect the taxonomic composition of diatoms but also determine their functional roles within ecosystems (Wu et al., 2019). For example, the study by Olenici et al. (2020) indicated that habitat differences can significantly influence diatom traits such as cell size, leading to spatial variation in functional traits Elevated nutrient concentrations, such as phosphorus (P) and nitrogen (N), tend to support the proliferation of larger, more motile diatom species, as these traits allow them to efficiently acquire resources and respond to environmental variability (Alipanah et al., 2018; Zheng et al., 2024). Essential soil ions, such as calcium and magnesium, can directly influence functional traits of diatoms by affecting cellular metabolism and structural stability (Lee et al., 2024). In our study, multiple regression analysis identified total phosphorus (TP) as the most significant factor influencing both taxonomic and functional diversity. RLQ and fourth-corner analyses further revealed that nutrients, including nitrate nitrogen (NO₃^−^-N), TP, and magnesium (Mg^2+^), had a significant effect on diatom motility (Figures 4a,b). Non-motile diatoms were positively correlated with TP, indicating that phosphorus-rich environments provide non-motile species with an advantage through efficient nutrient absorption. Additionally, weakly motile diatoms were positively correlated with Mg^2+^, likely due to the critical role of magnesium in metabolic processes that allow these species to perform optimally in moderately nutrient-rich environments. Highly motile diatoms showed a significant positive correlation with nitrate nitrogen (NO₃^−^-N), suggesting that these species rely on their motility to locate resources in nitrogen-enriched but spatially heterogeneous environments.

Therefore, soil physicochemical factors in the soil not only directly influence the taxonomic diversity of diatoms, but also regulate their functional traits, which further influence the functional diversity of the community. These results highlight the role of environmental filtering in selection of diatom functional traits across different ecological niches, supporting previous research on nutrient gradients and species functional adaptations. In aquatic-terrestrial ecotones, nutrient-rich areas promote the development of stronger motility and larger cells in diatoms to optimize resource utilization, while in nutrient-poor or harsh environments, diatoms adapt their structural traits to cope with environmental stress. This provides further evidence for understanding biodiversity and functional diversity in dynamic ecosystems.

### 4.3 The assembly mechanisms of diatom communities

The assembly mechanisms of diatom communities in the Danjiangkou Reservoir reflect a complex interplay between stochastic and deterministic processes, influenced by environmental variability in the aquatic-terrestrial ecotone (Hou et al., 2020). Sloan et al. (2006) demonstrated that in environments with high connectivity and low selective pressure, stochastic processes such as drift and dispersal limitation dominate. Similarly, Chase (2007) found that deterministic processes, including environmental filtering, prevail in stable or resource-limited environments, where species are selected based on their ability to adapt to stress, leading to niche differentiation. In fluctuating lake and wetland systems, hydrological variability tends to favor stochastic processes, while deterministic influences are stronger in drier gradients (Rojo, 2021; Hanson et al., 2012). Our results indicate that waterward zones are dominated by stochastic processes, with drift (60.54%) and dispersal limitation (30.30%) driving community assembly (Figure 5a), supporting neutral theory, which posits that frequent fluctuations cause niche overlap, promoting random colonization (Rojo, 2021). In contrast, landward zones are more influenced by deterministic processes due to environmental filtering from drought and nutrient stress, aligning with niche theory, where such stressors select for species with specific adaptations, fostering niche differentiation (Hanson et al., 2012). Studies by Wu et al. (2023) and Virta and Teittinen (2022) suggest that in hydrologically fluctuating systems, diatom communities are primarily shaped by stochasticity due to high dispersal potential and habitat connectivity, while Hu et al. (2023) and Zhao et al. (2024b) found that deterministic selection dominates in stable or resource-limited settings. Similarly, research in the Three Gorges Reservoir identified stronger heterogeneous selection in periodically dry areas, consistent with our observations in landward zones (Shi et al., 2021; Khurram et al., 2023). Overall, these findings align with the continuum concept of community assembly, where the balance between stochastic and deterministic forces shifts along environmental gradients. The coexistence of these mechanisms in ecotones highlights how hydrological variability and habitat heterogeneity jointly regulate diatom community dynamics. Understanding this balance builds a theoretical bridge between diatom ecology and broader ecological paradigms, providing insights into predicting how biodiversity patterns and community organization in reservoir ecosystems respond to hydrological regulation and environmental changes.

## 5 Limitations

The use of the 18S rRNA V4 region for taxonomic annotation enabled robust genus-level identification but has limitations in resolving species-level differences due to high sequence similarity among closely related diatom species. This may introduce uncertainties in species-level taxonomic assignments. Future studies could enhance accuracy by combining molecular markers like *rbcL* or *COI* with morphological analyses.

## 6 Conclusion

Our findings demonstrate that periodic flooding and drying events substantially modify soil physicochemical properties. This alteration subsequently impacts the abundance and functional traits of soil diatoms. Notably, distinct diatom communities were observed in the waterward and landward zones. Waterward areas tend to favor larger, motile species, whereas landward areas are dominated by smaller, less motile diatoms. Key environmental factors, particularly total phosphorus (TP), soil organic matter (SOM), and magnesium (Mg^2+^), exert a strong influence on diatom diversity and functional traits. Community assembly mechanisms differed between the zones due to environmental changes. In the waterward zone, stochastic processes, contributed by dispersal limitation, were more influential due to hydrological variability. In contrast, in the landward zone, deterministic processes such as environmental filtering, driven by harsher and more variable conditions, were dominant. This underscores how environmental gradients give rise to distinct community structures in dynamic ecotones. This research augments our understanding of how environmental variability in aquatic-terrestrial ecotones drives the structure and function of diatom communities. It offers crucial insights for managing biodiversity and ecosystem functions in reservoir systems, especially in the face of environmental changes such as nutrient loading and hydrological regulation.

## Data Availability

The data presented in the study are deposited in the NCBI repository (https://www.ncbi.nlm.nih.gov), accession number PRJNA1346901.
