# Supplementary material for: Diversity, functional traits and assembly processes of diatom community in the aquatic-terrestrial ecotone: a case study of Danjiangkou Reservoir, China
Source: Front Microbiol. 2025 Oct 31;16:1690275. doi: 10.3389/fmicb.2025.1690275 (PMC12616637; doi:10.3389/fmicb.2025.1690275)
Supplement: Supplementary file 1 [file Supplementary_file_1.docx]

**Community structure and assembly processes of diatom in the aquatic-terrestrial ecotone of Danjiangkou Reservoir**

Xucong Lyu^a^, Haiyan Chen^b,c^, Jialin Jing^a^, Huatao Yuan^a,b,*^, Jingxiao Zhang^a^, Xiaofei Gao^a^, Yunni Gao^a^, Xuejun Li^a,b,^^[[1]](#footnote-1)^

*^a^College of Fisheries, Henan Normal University, Xinxiang, Henan 453007, China*

*^b^Observation and Research Station on Water Ecosystem in Danjiangkou Reservoir of Henan Province, Nanyang, Henan 474450, China*

*^c^Ecological Environment Monitoring Center of Midline Project of South to North Water Division, Nanyang 474475, China*

Table S1 Sampling sites in the Danjiangkou Reservoir

| Name | Sample sites | Longitude | Latitude |
| --- | --- | --- | --- |
| Danjiang | DJC1 | 111.569716 | 32.861461 |
|  | DJC2 | 111.568591 | 32.858188 |
|  | DJC3 | 111.567079 | 32.855832 |
|  | DJF1 | 111.588212 | 32.864832 |
|  | DJF2 | 111.588212 | 32.864832 |
|  | DJF3 | 111.579836 | 32.855406 |
| Songgang | SGC1 | 111.623351 | 32.819912 |
|  | SGC2 | 111.631000 | 32.816403 |
|  | SGC3 | 111.637143 | 32.814165 |
|  | SGF1 | 111.639071 | 32.836440 |
|  | SGF2 | 111.644130 | 32.829982 |
|  | SGF3 | 111.644130 | 32.829982 |
| Nangang | NGC1 | 111.650979 | 32.754485 |
|  | NGC2 | 111.651282 | 32.752295 |
|  | NGC3 | 111.654700 | 32.749592 |
|  | NGF1 | 111.659691 | 32.758608 |
|  | NGF2 | 111.662475 | 32.754694 |
|  | NGF3 | 111.663137 | 32.751945 |
| Diaoshuikou | DSKC1 | 111.640343 | 32.716441 |
|  | DSKC2 | 111.639669 | 32.713806 |
|  | DSKC3 | 111.638448 | 32.709818 |
|  | DSKF1 | 111.652178 | 32.720571 |
|  | DSKF2 | 111.652093 | 32.716939 |
|  | DSKF3 | 111.648935 | 32.711100 |

Table S2 Diatom traits, categories, codes, and Detail description.

| Traits | Categories | Codes | Details |
| --- | --- | --- | --- |
| Cell Size(Berthon et al., 2011; Rimet and Bouchez, 2012) | Nano (0–100 μm^3^) | CellSize01 | Smaller cells (Nano and Micro) have higher nutrient uptake rates and growth rates, making them more resilient in nutrient-limited and high-disturbance environments, while larger cells (Macro and Large) tend to dominate in nutrient-rich, stable conditions but are less adaptable to environmental fluctuations. |
|  | Micro (100–300 μm^3^) | CellSize02 |  |
|  | Meso (300–600 μm^3^) | CellSize03 |  |
|  | Macro (600–1500 μm^3^) | CellSize04 |  |
|  | Large (≥ 1500 μm^3^) | CellSize05 |  |
| Biological Condition Gradient (BCG)(Hausmann et al., 2016) | Specialist species | BCG1 | Species range from highly specialized and sensitive (BCG1) to tolerant (BCG5), with BCG5 species thriving in disturbed or nutrient-rich environments, while BCG1 species are adapted to stable, low-nutrient conditions. |
|  | Highly sensitive species | BCG2 |  |
|  | Sensitive species | BCG3 |  |
|  | Indiscriminate species | BCG4 |  |
|  | Tolerant species | BCG5 |  |
| Motility(Passy, 2007) | Highly motile | Highly motile | Species with higher motility can actively move to access resources in changing environments, whereas non-motile species are adapted to stable environments where movement is unnecessary. |
|  | Moderately motile | Moderately motile |  |
|  | Slightly motile | Slightly motile |  |
|  | Weakly motile | Weakly motile |  |
|  | Non motile | Non motile |  |
| Attachment(Passy, 2007) | Prostrate | Prostrate | Species may attach to surfaces either vertically or prostrately, with stronger attachment providing stability in high-disturbance or low-nutrient environments. |
|  | Unattached | Unattached |  |
|  | Vertical | Vertical |  |
| Habitat(Wu et al., 2021) | Benthic | Benthic | Species may be benthic, living on the sediment surface and accessing substrate nutrients, or planktonic, floating in the water column and adapted to variable nutrient and light conditions. |
|  | Planktonic | Planktonic |  |
|  | Moist habitats | Moist habitats |  |
|  | Soils | Soils |  |

Table S3 Comparison of diatom traits between nearshore and inland areas

| Traits | Categories | Waterward | Landward |
| --- | --- | --- | --- |
|  | CellSize01 | 8.79±11.19a | 16.42±9.83a |
|  | CellSize02 | 29.88±20.36a | 26.26±19.28a |
| Cell Size | CellSize03 | 5.99±4.16a | 9.61±13.35a |
|  | CellSize04 | 7.64±6.62b | 31.40±37.78a |
|  | CellSize05 | 47.69±28.89a | 16.31±21.03b |
| Biological Condition Gradient (BCG) | BCG1 | 0.48±0.68a | 0.35±1.20a |
|  | BCG2 | 9.24±16.83a | 4.37±6.03a |
|  | BCG3 | 10.78±5.36a | 11.53±9.40a |
|  | BCG4 | 43.57±18.57a | 57.94±27.29a |
|  | BCG5 | 35.93±24.89a | 25.81±20.97a |
|  | Highly motile | 50.47±29.99a | 20.78±19.79b |
|  | Moderately motile | 23.48±15.56b | 57.29±28.99a |
| Motility | Slightly motile | 14.92±18.62a | 11.70±10.41a |
|  | Weakly motile | 0.41±0.56a | 1.25±2.61a |
|  | Non motile | 10.98±12.93a | 11.44±8.28a |
|  | Prostrate | 11.02±9.71a | 13.32±10.45a |
| Attachment | Unattached | 81.01±18.24a | 85.68±11.80a |
|  | Vertical | 8.00±15.52a | 1.00±1.84b |
|  | Benthic | 91.73±9.24a | 95.17±4.07a |
|  | Planktonic | 9.85±11.91a | 7.06±5.83a |
| Habitat | Moist habitats | 0.78±0.55b | 35.37±39.28a |
|  | Soils | 0.04±0.09b | 30.54±40.03a |

Note: Values are shown as the mean ± standard deviation (SD). Different letters indicate significant differences (Kruskal-Wallis test, *p* < 0.05)

Table S4 Trait Classification of Diatom Species

| **Species** | **Traits** | | | | |
| --- | --- | --- | --- | --- | --- |
|  | **code_size** | **Motility** | **Attachment** | **Habitat** | **BCG** |
| *Achnanthidium minutissimum* | CellSize01 | Slightly motile | Prostrate | Benthic | BCG3 |
| *Achnanthidium saprophilum* | CellSize01 | Slightly motile | Prostrate | Benthic | BCG4 |
| *Amphora* cf. | CellSize04 | Slightly motile | Prostrate | Benthic | BCG4 |
| *Amphora montana* | CellSize01 | Slightly motile , Moderately motile | Prostrate | Benthic | BCG4 |
| *Amphora* sp. | CellSize04 | Slightly motile | Prostrate | Benthic | BCG4 |
| *Achnanthidium daonense* | CellSize01 | Slightly motile | Prostrate | Benthic | BCG2 |
| *Campylodiscus levanderi* | CellSize05 | Highly motile | Unattached | Benthic | BCG5 |
| *Craticula accomoda* | CellSize02 | Moderately motile | Unattached | Benthic | BCG5 |
| *Craticula cuspidata* | CellSize05 | Highly motile | Unattached | Benthic | BCG4 |
| *Craticula pseudocitrus* | CellSize02 | Moderately motile | Unattached | Benthic | BCG5 |
| *Cymatopleura elliptica* | CellSize05 | Highly motile | Unattached | Benthic | BCG5 |
| *Cymatopleura solea* | CellSize05 | Highly motile | Unattached | Benthic | BCG4 |
| *Diadesmis gallica* | CellSize01 | Slightly motile | Unattached | Soils, Moist habitats, Benthic | BCG5 |
| *Diploneis parca* | CellSize01 | Moderately motile | Unattached | Moist habitats, Benthic | BCG3 |
| *Encyonopsis* sp. | CellSize02 | Slightly motile | Unattached | Moist habitats, Benthic | BCG2 |
| *Eolimna subminuscula* | CellSize01 | Weakly motile | Unattached | Benthic | BCG5 |
| *Epithemia turgida* | CellSize05 | Moderately motile | Prostrate | Benthic | BCG2 |
| *Fistulifera saprophila* | CellSize02 | Moderately motile | Unattached | Benthic | BCG5 |
| *Humidophila schmassmannii* | CellSize01 | Slightly motile | Unattached | Benthic | BCG5 |
| *Nitzschia dissipata* | CellSize02 | Highly motile | Unattached | Unattached | BCG3 |
| *Nitzschia draveillensis* | CellSize02 | Moderately motile | Unattached | Benthic | BCG4 |
| *Parlibellus delognei* | CellSize05 | Moderately motile | Unattached | Benthic | BCG5 |
| *Pauliella* sp. | CellSize02 | Slightly motile | Unattached | Moist habitats | BCG3 |
| *Surirella biseriata* | CellSize03 | Highly motile | Unattached | Benthic | BCG4 |
| *Surirella* cf. | CellSize03 | Highly motile | Unattached | Benthic | BCG4 |
| *Surirella* sp. | CellSize03 | Highly motile | Unattached | Benthic | BCG4 |
| *Tryblionella apiculata* | CellSize02 | Moderately motile | Unattached | Benthic | BCG5 |
| *Cocconeis pediculus* | CellSize03 | Slightly motile | Prostrate | Benthic | BCG4 |
| *Cocconeis placentula* | CellSize04 | Non-motile , Weakly motile | Prostrate | Benthic | BCG4 |
| *Cymbella excisa* | CellSize02 | Slightly motile | Vertical | Benthic | BCG2 |
| *Cymbella* sp. | CellSize02 | Slightly motile | Vertical | Benthic | BCG2 |
| *Diatoma tenuis* | CellSize02 | Non-motile | Unattached, Prostrate | Benthic, Planktonic | BCG2 |
| *Encyonema minutum* | CellSize02 | Slightly motile | Unattached | Benthic | BCG3 |
| *Entomoneis Surirella* sp. | CellSize05 | Highly motile | Unattached | Benthic | BCG4 |
| *Fistulifera pelliculosa* | CellSize01 | Non-motile | Prostrate | Benthic | BCG2 |
| *Mayamaea atomus* | CellSize01 | Weakly motile | Unattached | Benthic | BCG3 |
| *Gomphonema angustum* | CellSize03 | Moderately motile | Vertical | Benthic | BCG2 |
| *Gomphonema clevei* | CellSize02 | Moderately motile | Vertical | Benthic | BCG3 |
| *Gomphonema* sp. | CellSize02 | Moderately motile | Vertical | Benthic | BCG3 |
| *Gyrosigma acuminatum* | CellSize05 | Highly motile | Unattached | Benthic | BCG4 |
| *Hantzschia amphioxys* | CellSize04 | Moderately motile | Unattached | Soils, Moist habitats, Benthic | BCG4 |
| *Hantzschia sigma* | CellSize04 | Moderately motile | Unattached | Soils, Moist habitats, Benthic | BCG4 |
| *Luticola permuticopsis* | CellSize02 | Moderately motile | Unattached | Soils, Moist habitats, Benthic | BCG5 |
| *Luticola ventricosa* | CellSize02 | Moderately motile | Unattached | Soils Moist habitats Benthic | BCG5 |
| *Hippodonta capitata* | CellSize02 | Moderately motile | Unattached | Benthic | BCG4 |
| *Navicula arenaria* | CellSize01 | Moderately motile | Unattached | Benthic | BCG4 |
| *Navicula cryptocephala* | CellSize02 | Moderately motile | Unattached | Benthic | BCG4 |
| *Navicula cryptotenelloides* | CellSize02 | Highly motile | Unattached | Benthic | BCG5 |
| *Navicula phyllepta* | CellSize03 | Moderately motile | Unattached | Benthic | BCG4 |
| *Navicula reinhardtii* | CellSize04 | Moderately motile | Unattached | Benthic | BCG3 |
| *Navicula* sp. | CellSize03 | Moderately motile | Unattached | Benthic | BCG4 |
| *Neidium productum* | CellSize04 | Moderately motile | Unattached | Benthic | BCG1 |
| *Nitzschia amphibia* | CellSize02 | Moderately motile | Prostrate | Benthic | BCG5 |
| *Nitzschia communis* | CellSize02 | Moderately motile | Prostrate | Benthic | BCG5 |
| *Nitzschia Nitzschia dissipata* | CellSize02 | Highly motile | Unattached | Benthic | BCG3 |
| *Nitzschia filiformis* | CellSize03 | Moderately motile | Unattached | Benthic | BCG4 |
| *Nitzschia fonticola* | CellSize02 | Moderately motile | Prostrate | Benthic | BCG4 |
| *Nitzschia hantzschiana* | CellSize01 | Moderately motile | Prostrate | Benthic | BCG4 |
| *Nitzschia inconspicua* | CellSize01 | Moderately motile | Unattached | Benthic | BCG4 |
| *Nitzschia palea* | CellSize01 | Moderately motile | Prostrate | Benthic | BCG5 |
| *Nitzschia pusilla* | CellSize02 | Moderately motile | Prostrate | Benthic | BCG5 |
| *Nitzschia sigma* | CellSize04 | Moderately motile | Unattached | Benthic | BCG4 |
| *Nitzschia* sp. | CellSize01 | Highly motile | Unattached | Benthic | BCG2 |
| *Caloneis budensis* | CellSize02 | Slightly motile | Unattached | Benthic | BCG3 |
| *Caloneis lewisii* | CellSize03 | Moderately motile | Unattached | Benthic | BCG4 |
| *Pinnularia* cf. | CellSize04 | Moderately motile | Unattached | Benthic | BCG3 |
| *Pinnularia subgibba* | CellSize04 | Moderately motile | Unattached | Benthic | BCG3 |
| *Pinnunavis* sp. | CellSize04 | Moderately motile | Unattached | Benthic | BCG3 |
| *Planothidium caputium* | CellSize01 | Slightly motile | Unattached | Benthic | BCG4 |
| *Planothidium frequentissimum* | CellSize01 | Slightly motile | Unattached | Benthic | BCG4 |
| *Eolimna minima* | CellSize01 | Slightly motile | Unattached | Moist habitats Benthic | BCG4 |
| *Sellaphora bacillum* | CellSize03 | Moderately motile | Prostrate | Benthic | BCG2 |
| *Sellaphora capitata* | CellSize02 | Moderately motile | Unattached | Benthic | BCG3 |
| *Sellaphora* cf. | CellSize01 | Moderately motile | Unattached | Benthic | BCG3 |
| *Sellaphora minima* | CellSize01 | Moderately motile | Unattached | Benthic | BCG3 |
| *Sellaphora pupula* | CellSize03 | Moderately motile | Unattached | Benthic | BCG3 |
| *Sellaphora seminulum* | CellSize01 | Moderately motile | Unattached | Benthic | BCG3 |
| *Sellaphora* sp. | CellSize01 | Moderately motile | Unattached | Benthic | BCG3 |
| *Surirella splendida* | CellSize05 | Highly motile | Unattached | Benthic | BCG3 |
| *Aulacoseira granulata* | CellSize02 | Non-motile | Unattached | Planktonic | BCG5 |
| *Aulacoseira subarctica* | CellSize01 | Non-motile | Unattached | Planktonic | BCG3 |
| *Fragilaria capucina* | CellSize01 | Non-motile | Unattached | Benthic | BCG3 |
| *Fragilaria rumpens* | CellSize01 | Non-motile | Prostrate | Benthic | BCG3 |
| *Fragilaria vaucheriae* | CellSize01 | Non-motile | Vertical | Benthic | BCG3 |
| *Pseudostaurosiropsis* sp. | CellSize01 | Non-motile | Prostrate | Benthic | BCG3 |
| *Opephora guenter grassii* | CellSize01 | Weakly motile | Unattached | Benthic | BCG3 |
| *Stauroforma rinceana* | CellSize01 | Non-motile | Unattached | Benthic | BCG2 |
| *Staurosira* sp. | CellSize01 | Non-motile | Vertical | Benthic Planktonic | BCG4 |
| *Staurosirella* sp. | CellSize02 | Non-motile | Unattached | Benthic Planktonic | BCG3 |
| *Synedra berolinensis* | CellSize01 | Non-motile | Unattached | Planktonic | BCG2 |
| *Synedra angustissima* | CellSize02 | Non-motile | Unattached | Planktonic | BCG2 |
| *Ulnaria ulna* | CellSize04 | Non-motile | Prostrate | Benthic Planktonic | BCG2 |
| *Cyclotella cryptica* | CellSize02 | Non-motile | Unattached | Planktonic | BCG5 |
| *Cyclotella meneghiniana* | CellSize02 | Non-motile | Unattached | Planktonic | BCG5 |
| *Cyclotella* sp. | CellSize02 | Non-motile | Unattached | Planktonic | BCG5 |
| *Acanthoceras* sp. | CellSize02 | Non-motile | Unattached | Planktonic | BCG4 |
| *Discostella woltereckii* | CellSize01 | Non-motile | Planktonic | Planktonic | BCG4 |
| *Lindavia radiosa* | CellSize03 | Non-motile | Unattached | Planktonic | BCG4 |
| *Pleurosira* cf. | CellSize05 | Non-motile | Prostrate | Benthic | BCG5 |
| *Stephanodiscus niagarae* | CellSize05 | Non-motile | Unattached | Planktonic | BCG4 |
| *Conticribra weissflogii* | CellSize02 | Non-motile | Unattached | Planktonic | BCG4 |
| *Geissleria decussis* | CellSize02 | Moderately motile | Unattached | Benthic | BCG4 |


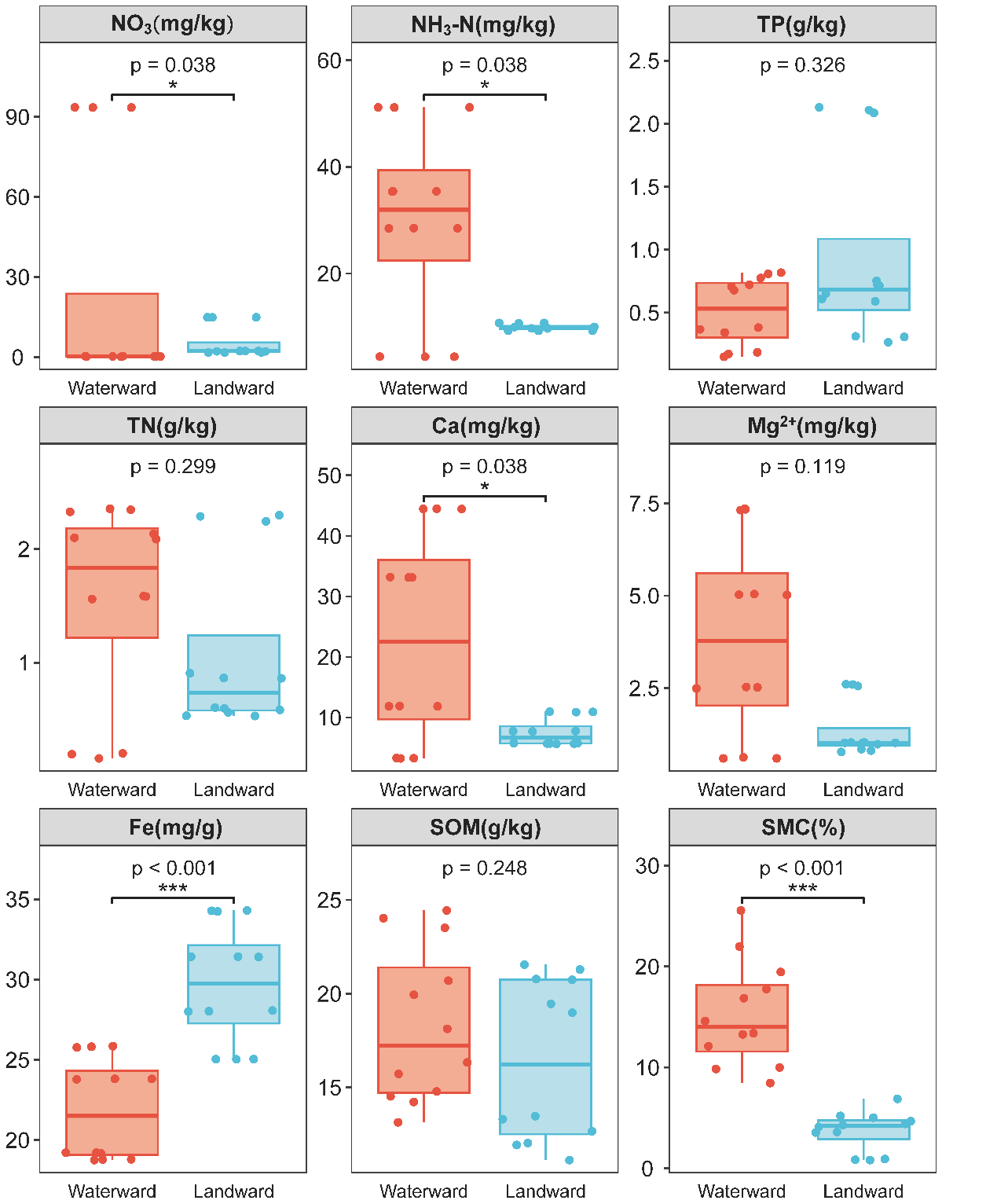


Fig. S1 Boxplot of soil environmental factors for waterward and landward sites. The Kruskal-Wallis test was used to assess differences between different sites, **p* < 0.05, ***p*<0.01, ****p*<0.001.


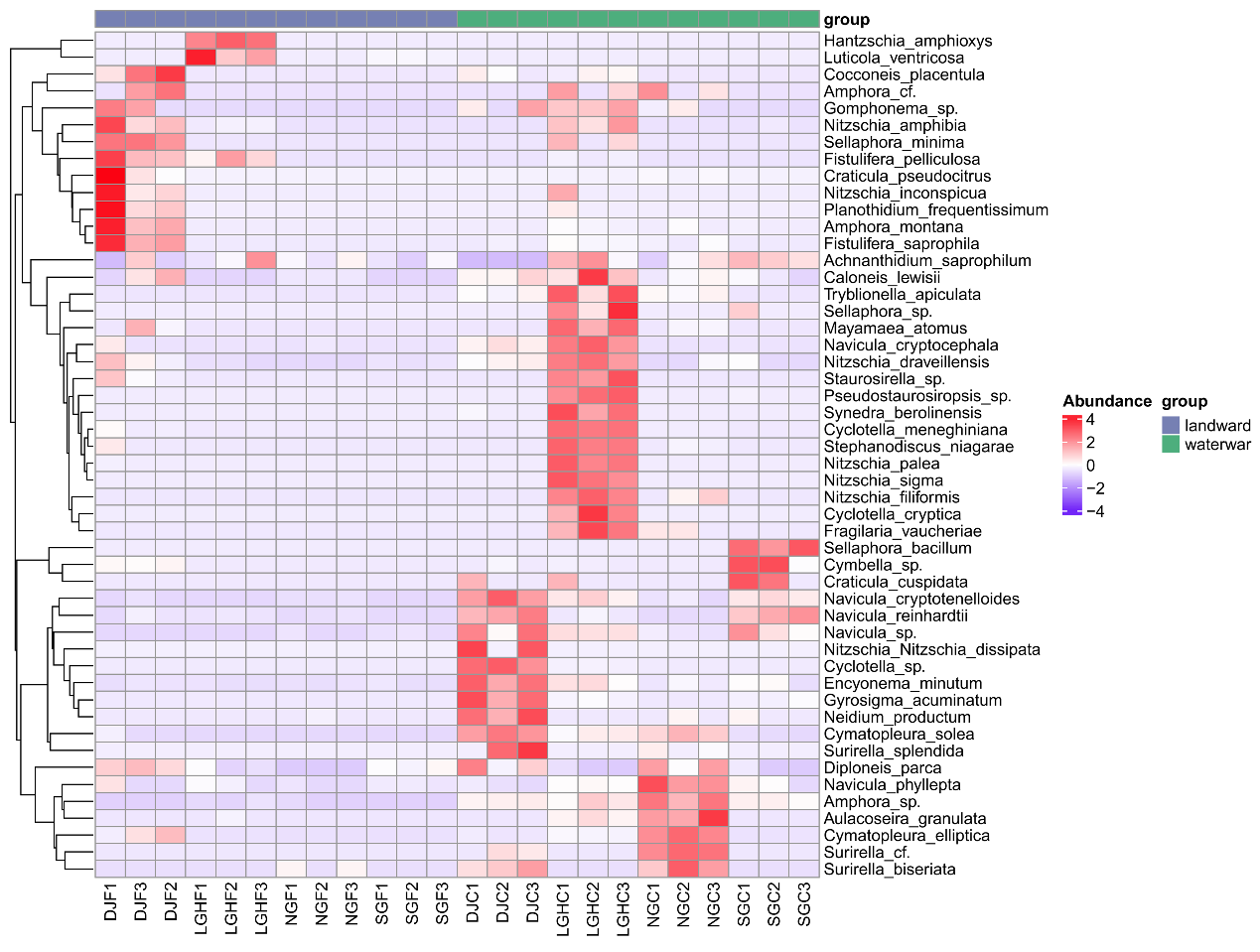


Fig. S2 Heatmap of Diatom Species Composition Clustering Analysis in Waterward and Landward Zones. The data were normalized using Z-score.


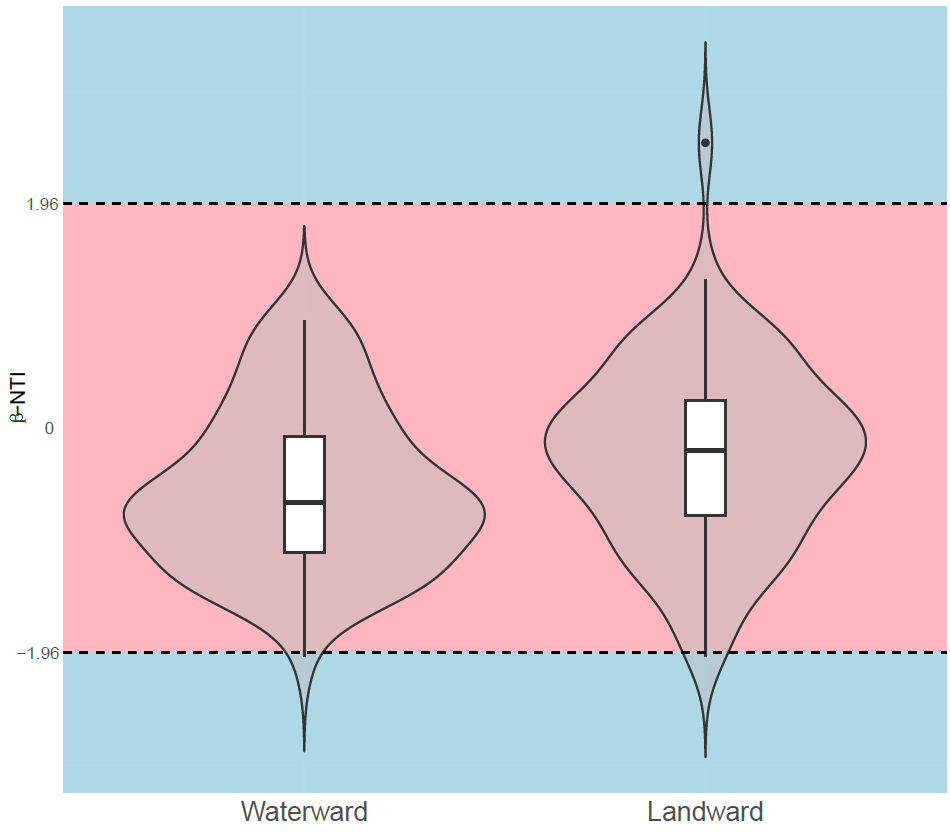


Fig. S3 Boxplot and violin plot showing the β-NTI distribution for Waterward and Landward samples. The red area where |βNTI| < 1.96 represents neutral processes, while the blue area where |βNTI| > 1.96 indicates deterministic processes.

**References**

Berthon, V., Bouchez, A., Rimet, F., 2011. Using diatom life-forms and ecological guilds to assess organic pollution and trophic level in rivers: a case study of rivers in south-eastern France. Hydrobiologia 673, 259-271.

Hausmann, S., Charles, D.F., Gerritsen, J., Belton, T.J., 2016. A diatom-based biological condition gradient (BCG) approach for assessing impairment and developing nutrient criteria for streams. Sci Total Environ 562, 914-927.

Passy, S.I., 2007. Diatom ecological guilds display distinct and predictable behavior along nutrient and disturbance gradients in running waters. Aquatic Botany 86, 171-178.

Rimet, F., Bouchez, A., 2012. Life-forms, cell-sizes and ecological guilds of diatoms in European rivers. Knowl Manag Aquat Ec.

Wu, N., Zhou, S., Zhang, M., Peng, W., Guo, K., Qu, X., He, F., 2021. Spatial and local environmental factors outweigh geo‐climatic gradients in structuring taxonomically and trait‐based β‐diversity of benthic algae. J Biogeogr 48, 1842-1857.

1. Corresponding authors at: Henan Normal University, Xinxiang 453007, China.

   *Email address*: yuanhuatao@htu.edu.cn(H. Yuan), xjli@htu.cn (X. Li). [↑](#footnote-ref-1)
